# A lifestyle intervention supported by mobile health technologies to improve the cardiometabolic risk profile of individuals at risk for cardiovascular disease and type 2 diabetes: study rationale and protocol

**DOI:** 10.1186/1471-2458-13-1051

**Published:** 2013-11-07

**Authors:** Melanie I Stuckey, Sheree Shapiro, Dawn P Gill, Robert J Petrella

**Affiliations:** 1 Lawson Health Research Institute, Aging Rehabilitation and Geriatric Care Research Centre, 801 Commissioners Rd E, Ste B 3002, London, Ontario N6C 5J1, Canada; 2 Faculty of Health Sciences, University of Western Ontario, London Ontario, Canada; 3 School of Public Health, University of Washington, Seattle WA, USA; 4 Schulich School of Medicine and Dentistry, University of Western Ontario, London Ontario, Canada

**Keywords:** Mobile health, Metabolic syndrome, Exercise prescription, Exercise intervention, Disease prevention, Rural health

## Abstract

**Background:**

Metabolic syndrome is a cluster of cardiovascular risk factors that greatly increase the risk of developing cardiovascular disease and type 2 diabetes. Regular exercise improves the risk profile, but most people do not successfully change their exercise habits to beneficially reduce risk. Tailored exercise prescribed by a family physician has shown promise as a means to increase fitness and reduce cardiometabolic risk, but optimal implementation practices remain unknown. Mobile health technologies have proved to be a beneficial tool to achieve blood pressure and blood glucose control in patients with diabetes. These technologies may address the limited access to health interventions in rural and remote regions. However, the potential as a tool to support exercise-based prevention activities is not well understood. This study was undertaken to investigate the effects of a tailored exercise prescription alone or supported by mobile health technologies to improve metabolic syndrome and related cardiometabolic risk factors in rural community-dwelling adults at risk for cardiovascular disease and type 2 diabetes.

**Methods/Design:**

Adults (*n* = 149) with at least two metabolic syndrome risk factors were recruited from rural communities and randomized to either: 1) an intervention group receiving an exercise prescription and devices for monitoring of risk factors with a smartphone data portal equipped with a mobile health application; or 2) an active control group receiving only an exercise prescription. All participants reported to the research centre at baseline, and at 12-, 24- and 52-week follow-up visits for measurement of anthropometrics and blood pressure and for a blood draw to test blood-borne markers of cardiometabolic health. Vascular and autonomic function were examined. Fitness was assessed and exercise prescribed according to the Step Test and Exercise Prescription protocol.

**Discussion:**

This study tested the effects of a prescriptive exercise intervention alone, versus one supported by mobile health technology on cardiometabolic risk factors. The intervention was designed to be translated into clinical or community-based programming. Results will contribute to the current literature by investigating the utility of mobile health technology support for exercise prescription interventions to improve cardiometabolic risk status and maintain improvements over time; particularly in rural communities.

**Trial registration:**

Clinical trials registration: NCT01944124

## Background

Cardiovascular disease is the leading cause of death worldwide accounting for 48% of mortality and 10% of global disease burden [1]. Cardiovascular complications are common in patients with type 2 diabetes mellitus, with heart disease responsible for 68% and stroke responsible for 16% of mortality in this patient population in the United States [2]. Metabolic syndrome is a defined set of risk factors including elevated blood pressure, dysglycemia, dyslipidemia and abdominal obesity, which increases the risk of developing cardiovascular diseases and type 2 diabetes mellitus [3,4]. Metabolic syndrome risk factors often present concurrently with other global cardiometabolic risk factors [4], ranging from standard clinical variables, such as low density lipoprotein and total cholesterol, high sensitivity C-reactive protein and body mass index to novel physiologic factors requiring specialized testing, such as vascular and autonomic function. While certain risk factors have been identified, it remains unclear which variables may be clinically important as early risk markers for cardiovascular complications. Early identification and management of these risk factors may be an important strategy for preventing cardiovascular diseases and type 2 diabetes mellitus.

Metabolic syndrome is highly treatable and lifestyle changes, including increased physical activity and regular exercise are recommended as first line treatment [4]. Longitudinal studies have shown that adoption of aerobic exercise training in healthy and sedentary middle-aged adults with metabolic syndrome improved the cardiometabolic risk profile and reduced the prevalence of metabolic syndrome [5-8]. Improved lifestyle behaviours in patients with metabolic syndrome are important to prevent the progression to chronic diseases, such as type 2 diabetes mellitus. The Diabetes Prevention Program tested an intensive lifestyle intervention with the goal of seven percent weight loss through a low-calorie, low-fat diet along with participation in a minimum of 150 minutes of moderate-to-vigorous physical activity per week [9]. This lifestyle intervention was more effective in preventing type 2 diabetes mellitus than pharmacological therapy with metformin or placebo plus standard lifestyle recommendations in overweight adults with impaired glucose tolerance [9]. At an average follow-up of 2.8 y, incident diabetes was 31% less in the metformin group and 58% less in the intensive lifestyle intervention group compared to the placebo group [9]. These studies highlight the importance of physical activity and exercise to manage risk and prevent progression to chronic disease.

Despite the known benefits of exercise, accelerometer data showed that 85% of Canadians [10] and 90% of Americans [11] do not meet physical activity guidelines. Exercise prescription in primary care may be an effective strategy to increase physical activity [12-14]. A systematic review concluded that exercise prescription interventions were effective at increasing physical activity, especially when they included a written prescription, considered behaviour change strategies and provided training and materials for physicians [14]. Despite known benefits, implementation in primary care remains low due to a gap in our understanding of how to best target and deliver individualized exercise prescriptions to patients at risk in the community.

In recent years, mobile technologies have been used as a medium for implementing health monitoring interventions. Mobile health has been used as a tool to support patient management of chronic diseases including type 2 diabetes mellitus [15] and hypertension [16-18], but little attention has been paid to leveraging mobile health technologies for risk management for disease prevention. To date, one pilot study examined the effects of a cardiovascular disease prevention intervention using an electronic health intervention with a mobile health component to manage metabolic syndrome risk factors [19]. The workplace electronic health program consisted of a four-week education module (60 minutes per session) plus access to telephone counseling, short message service messages and/or email messages for six months. Body fat, blood pressure and pedometer steps were regularly monitored. Following six months, waist circumference, systolic and diastolic blood pressure and triglycerides were reduced, with no changes in fasting plasma glucose or high density lipoprotein cholesterol [19]. This six-month eHealth intervention improved metabolic syndrome risk, but it was limited to a population of workers whose employers accepted the Healthy Workplace Program. Additionally, fasting plasma glucose was not monitored despite evidence from interventions in patients with type 2 diabetes mellitus supporting home-monitoring as an effective tool for improving fasting plasma glucose [15].

Access to healthcare is often reduced in rural compared to urban communities. For example, Huron County in rural southwestern Ontario has a 27.5% physician vacancy rate (number of positions available for general practitioners) compared to 0% vacancy rate in nearby urban centres [20]. Innovative technologies including mobile health monitoring have the potential to increase access to healthcare. Our group completed a pilot study testing the feasibility and utility of deploying a mobile health technology kit for self-management of metabolic syndrome risk factors and physical activity in a rural population [21,22]. Twenty-four participants aged 30-72 years monitored their blood pressure three times per week and blood glucose two times per day with Bluetooth™ enabled devices paired with a smartphone. Additionally, pedometer steps were manually inputted daily and body weight was inputted weekly [21,22]. A health monitoring system including an application on the smartphone and a secure online database was used to transmit and store data [21]. Alarms were triggered when measurements were outside of pre-determined limits. Following the eight-week intervention, waist circumference, diastolic blood pressure and total cholesterol were reduced, physical activity and fitness were increased [22], and autonomic function was improved [23]. Furthermore, participants reported that the technology assisted in adopting new practices to improve wellbeing and did not take too much time or interfere with activities of daily living [21]. This study, however, could not conclude whether the mobile health protocol had added benefit to the exercise prescription.

Therefore, the purpose of this trial was to investigate the effects of a mobile health supported exercise prescription, compared to exercise prescription alone on metabolic syndrome and other global cardiometabolic risk factors in individuals at risk for cardiovascular disease and type 2 diabetes. It was hypothesized that the primary outcome, systolic blood pressure, and secondary outcomes would be improved in both groups, but to a greater extent in the mobile health intervention group at 12 weeks and that these changes would be better maintained at 24 and 52 weeks in the intervention group with mobile health support compared with the active control group.

## Methods/Design

### Study design

This study was a randomized controlled trial with two intervention arms: 1) the intervention group, which received a tailored exercise prescription and a mobile health technology kit; and 2) the active control group, which received only a tailored exercise prescription. Adults aged 18-70 years were screened for inclusion. Participants (*n* = 149) presenting with at least two metabolic syndrome risk factors reported to the clinic (Gateway Rural Health Research Centre, Seaforth, Ontario, Canada) at baseline (V0) and for 12- (V1), 24- (V2) and 52-week (V3) follow-up visits.

#### *Recruitment and screening process*

This study was approved by the University of Western Ontario Research Ethics Board (Protocol #15828). Community-dwelling adults aged 18-70 years were recruited via newspaper or radio advertisement, flyers, referral from healthcare professional and word of mouth. Interested candidates contacted the study coordinator for telephone screening. Participants were eligible if they had two or more metabolic syndrome risk factors according to National Cholesterol Education Program Adult Treatment Panel III criteria: waist circumference ≥ 88 cm (women) or ≥ 102 cm (men); systolic blood pressure ≥ 135 mmHg and/or diastolic blood pressure ≥ 85 mmHg; fasting plasma glucose ≥ 6.1 mmol/L; triglycerides ≥ 1.7 mmol/L; and high density lipoprotein cholesterol ≤ 1.29 mmol/L (women) or ≤ 1.02 mmol/L (men) [24]. Exclusion criteria were: systolic blood pressure > 180 mmHg and/or diastolic blood pressure > 110 mmHg; type 1 diabetes; history of myocardial infarction, angioplasty, coronary artery bypass or cerebrovascular ischemia/stroke; symptomatic congestive heart failure; atrial flutter; unstable angina; unstable pulmonary disease; use of medications known to affect heart rate; second or third degree heart block; history of alcoholism, drug abuse or other emotional cognitive or psychiatric problems; pacemaker; unstable metabolic disease and orthopedic or rheumatologic problems that could impair the ability to exercise. Participants who passed the initial screening were booked for a V0 appointment. To facilitate scheduling of group mobile health technology training sessions, block randomization was used, such that all participants attending appointments during week one were allocated to the intervention group and all participants attending appointments during week two were allocated to the control group and so on.

### Final eligibility screening at baseline visit

Participants who qualified for the study reported to the clinic following an eight hour fast. Participants were instructed to abstain from caffeinated or alcoholic beverages for 12 hours and intense physical activity for 24 hours prior to their study visits. Voluntary informed consent was obtained from all participants. Inclusion and exclusion criteria were verified by a medical history interview and by measuring metabolic syndrome risk factors.

### Laboratory procedures for baseline and follow-up visits

#### *Demographics and medical history*

During the baseline visit only, participant demographics recorded included: date of birth, sex, race and postal code. An interview was then conducted to determine medical history, which included documenting information regarding smoking status, alcohol consumption, history of heart/vascular disease, history of endocrine disease, gynecological history, lifestyle habits, past illnesses and surgeries, and current medications. While the full interview was conducted only at baseline, it was requested that participants contact the study coordinator should there be any change in any of these items and at each follow-up visit participants were asked if there was any change in medication or health status.

#### *Physical examination*

At each study visit, anthropometrics were measured. Body weight was measured with an automated scale and height was measured with a standard stadiometer in light clothing with shoes removed. Waist circumference was measured (to the nearest 0.5 cm) following a normal exhalation at the mid-point between the twelfth rib and superior boarder of the iliac crest [25]. Blood pressure was measured with an automated device (BPTru®, Coquitlam, Canada) in the supine position following a five-minute rest period. The average of the last two of three measures was used to determine resting blood pressure (to the nearest 1 mmHg). Participants continued to rest supine for an additional 30 mins after which research personnel certified in venipuncture drew blood from the anticubital vein. Blood was sent to a central laboratory (GammaDyna Care, London, Ontario) for analysis of fasting plasma glucose, lipid profile, glycated hemoglobin, insulin, high sensitivity C-reactive protien, catecholamines, and estrogen.

#### *Vascular and autonomic testing*

Upon completion of the venipuncture procedure, a standardized snack was consumed. Participants were instrumented for collection of continuous R-R intervals with a lead II electrocardiogram (Colin Pilot 9200, Colin Medical Instruments, San Antonio, Texas) and respiratory rate by belt transducer (Pneumotrace II, ADInstruments, Colorado Springs, Colorado) secured around the thorax. Data were collected during ten minutes of supine rest, with external stimuli such as light and noise controlled to ensure signal stability. All measures were sampled at 1000 Hz, input into a data acquisition board (PowerLab ML795, ADInstruments) for analog-to-digital signal conversion with LabChart7Pro software (ADInstruments) and stored for offline analysis.

The left carotid and brachial arteries were imaged longitudinally just proximal to the carotid sinus and anticubital fossa, respectively, using B mode ultrasound (VingMed System 5, GE Ultrasound A/S, Horton, Norway). The average of three measures of each systolic and diastolic diameter and intima media thickness were recorded for each site. A sphygmomanometer blood pressure cuff was then secured on the left forearm just distal to the elbow for assessment of flow mediated dilation [26]. The brachial artery was imaged as described above. The cuff was inflated to 200 mmHg for five minutes and then deflated to induce reactive hyperemia. Beat-by-beat flow and continuous brachial arterial diameter were recorded with Doppler ultrasound for five minutes following the release of the pressure cuff.

#### *Fitness assessment and exercise prescription*

The Step Test and Exercise Prescription (STEP™) protocol was used for fitness assessment and exercise prescription. This tool is endorsed by the College of Family Physicians of Canada as part of accredited Continuing Health Education curriculum for improving physical activity education among family physicians. It has been validated in adults aged 18-65 years, (Unpublished observations) and 65-85 years [27]. In adults aged 18-65 years, the fitness assessment was shown to overestimate fitness by 7 ml/kg/min [Unpublished observations]. However, since STEP™ results (i.e. aerobic fitness) are used as a motivational tool, its use has been recommended for counselling and prescription purposes, but not as a primary outcome measure [Unpublished observations]. STEP™ has proved useful as a tool in primary care to increase physical activity and improve metabolic syndrome and global cardiometabolic risk factors [28].

**Figure 1:**
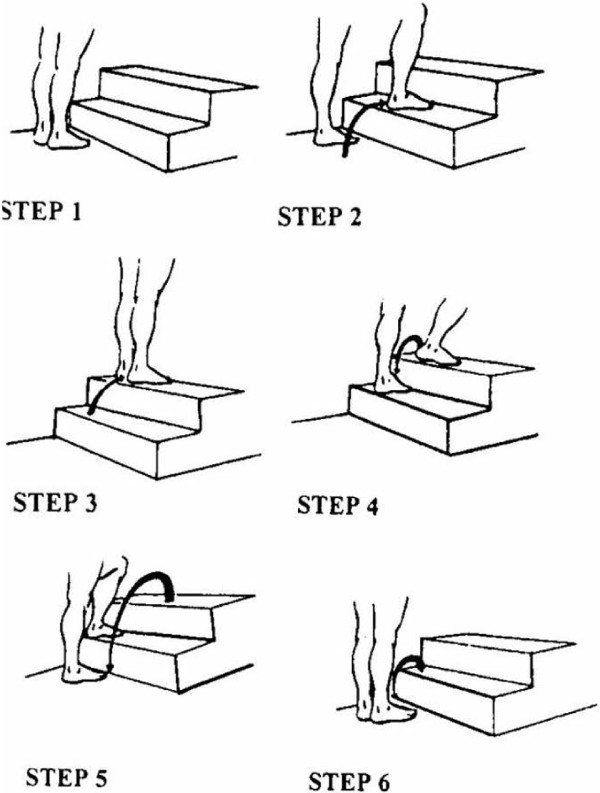
Step Test and Exercise Prescription (STEP™) protocol.

The STEP™ test was conducted by a research assistant with training in kinesiology. Resting heart rate was measured by palpation of the radial artery for 10 seconds. Participants were instructed to step up and down a set of two steps (each with a 20 cm rise) twenty times at a pace considered normal for climbing stairs (Figure 1). Upon completion of the test, post-exercise heart rate was palpated from the radial artery for a 10-second count. Time to complete the test (in seconds) and post-exercise heart rate (in beats per minute; bpm) were entered into an equation to estimate fitness (maximal oxygen uptake) [28]. Fitness was classified as poor, fair, good or excellent based on age, sex and maximal oxygen uptake (Additional file 1).

The same research assistant who conducted the STEP™ test counselled the participant and prepared an individualized exercise prescription based on fitness and participant preferences. Participants worked with the research assistant to identify three personal fitness goals. Additionally, barriers to exercise or lifestyle changes were identified and strategies to overcome these barriers were developed. The final exercise prescription was based on American College of Sports Medicine guidelines [29] and included the frequency, intensity, type and duration of exercise. Frequency was ideally set at (or increased to) most days of the week. Training heart rate was calculated as a percentage of the age predicted maximum heart rate (220-age) and was based on fitness classification from the STEP™ fitness assessment with 70, 75, 80 and 85% of predicted maximum heart rate prescribed for participants with poor, fair, good and excellent fitness, respectively. The type of exercise most commonly prescribed was walking, but other aerobic activities were also chosen including, but not limited to, aerobic exercise classes, swimming, cycling and team sports. Resistance exercises were also prescribed one to three days per week, depending on fitness, and consisted of exercises such as squats, calf raises and push ups. When participants had equipment available to them for resistance exercise, programs were designed specific to their needs. All participants were encouraged to accumulate a minimum of 150 minutes per week of moderate-to-vigorous intensity physical activity according to global guidelines [30]. Exercise bouts could be a minimum of 10 minutes with the goal of increasing time to 30 minutes per session or more. Since participants in the intervention group received a pedometer, step goals were also included in their prescription [31].

Participants in the active control group were given a journal to log their activity daily including exercise heart rate. The intervention group logged their activity with their mobile health monitoring application (see below).

### Behavioural and health status questionnaires

Participants completed the Short Form-36 questionnaire [32]. Raw scores were transformed using the guidelines developed by Medical Outcomes Trust. Eight dimensions of quality of life were examined: physical functioning; role-physical; role-emotional; bodily pain; vitality; mental health; social functioning; and general health. Raw scores were converted to a score out of 100 for each dimension, with 0 and 100 representing an extremely poor and extremely good quality of life, respectively. The SF-36 has proved to be valid and reliable [33,34]. Dietary habits, exercise, blood glucose testing, foot care and smoking habits were recorded using the Summary of Diabetes Self-Care Activities survey, which is a brief, reliable and valid measure of these lifestyle behaviours [35].

The Decisional Balance scale was used to assess perceptions of pros and cons to participating in exercise [36] and the Exercise Self-efficacy scale was used to assess confidence in participating in an exercise program [37].

A survey assessing current technology use, comfort with and burden of technology was administered at each visit to the intervention group. The active control group also completed a survey at V3. This survey was included solely to receive feedback regarding the technology, which would be used to modify the protocol for future studies.

### Mobile health technology

In addition to the exercise prescription, the intervention group also received a mobile health technology kit for self-monitoring of biometrics and physical activity. We previously tested the feasibility of delivering this intervention to this population and showed that the technology was accepted and motivational to participants in the pilot study [21]. The kit included a smartphone (Blackberry® Curve 8300 or 8530) equipped with Healthanywhere health monitoring application (Biosign Technologies Inc., Markham, Ontario, Canada), a Bluetooth™ enabled blood pressure monitor (A & D Medical, UA-767PBT, San Jose, California, USA), a glucometer (Lifescan One Touch Ultra2™, Milpitas, California, USA) with Bluetooth™ adapter (Polymap Wireless, PWR-08-03, Tucson, Arizona, USA) and a pedometer (Omron, HJ-150, Kyoto, Japan). All participants were required to attend a group training session (approximately two-hour duration) following V0. Those who could not attend group training sessions were given an individual training session at the end of their baseline visit. During the training session, participants were instructed on proper use of devices and techniques to ensure proper measurements. Participants were given the opportunity to practice taking measurements, to ask any questions related to the mobile health protocol and were provided with a telephone number and email address of study personnel for troubleshooting.

Throughout the intervention period, home blood pressure and fasting plasma glucose were to be submitted thrice weekly upon waking. Blood pressure was to be taken in the seated position with feet flat on the floor, back supported and arm supported at heart height. Blood pressure was measured once following five minutes of seated rest. Fasting plasma glucose was measured prior to consumption of food or drink. The pedometer was to be attached to the waistband at the front of the hip daily upon waking and removed just before bed (or during activities that could damage the pedometer, such as swimming and contact sports). Pedometer steps were input nightly. Home body weight was measured monthly with consistent timing and equipment. All submitted measures were date and time stamped and transmitted in real time to a secure database that was monitored by research personnel. Thresholds were set for systolic blood pressure (60-220 mmHg), diastolic blood pressure (40-110 mmHg) and fasting plasma glucose (3.0-15.0 mmol/L). Submitted readings that were outside these limits triggered an alarm that was sent to the study physician’s smartphone for immediate follow-up. Trends for consistently increasing measures were monitored by research assistants and brought to the study physician’s attention as necessary for follow-up.

Exercise was logged with the Healthanywhere activity tracking module. An individualized list of activities and target heart rate from the participant’s exercise prescription was compiled, and the participant was to select the completed activity and heart rate achieved following each planned exercise session.

Participants received a heart rate monitor (Suunto Memory Belt, Vantaa, Finland), which was secured around the chest on three occasions during the week following each visit. On the least active day of the week, participants were to wear the monitor for a 24-hour period and on two separate occasions they were to perform an orthostatic challenge with five minutes each of supine rest, seated rest and quiet standing. Heart rate monitors were returned to the clinic and data were downloaded to a personal computer with Suunto Training Manager software (Version 2.3.0).

Details of security for data transfer and database storage has been reported elsewhere [21]. Briefly, real-time data were encrypted and transmitted from the smartphone to the server and database by secure internet protocol. Biometric data were deidentified for transmission and linked to participant data once on the protected server. Database access was limited to authorized researchers. Healthanywhere accounts were controlled and protected remotely so that in the case of device loss, the account could be deactivated to prevent unauthorized access to participant health information.

### Data processing

#### *Mobile health*

Deidentified health data will be downloaded from the Healthanywhere database for offline analysis. Compliance to the self-monitoring protocol will be calculated as the percentage of measurements that were completed overall and for each device. The average of monthly measurements will be calculated and used to examine change in mobile health biometric and physical activity measures over time. Data from mobile health exercise logs will be processed the same as paper logs, described below.

#### *Exercise log data*

Entries from exercise logs will be input to a personal computer and coded according to exercise type. Exercise data will be used for descriptive purposes only and exercise characteristics such as frequency, duration and intensity were documented. Compliance to the exercise prescription will be examined by calculating the percentage of exercise sessions completed.

#### *Heart rate variability processing*

Data files from electrocardiogram collection of R-R interval in the clinic in all participants and home monitoring of R-R interval for 24 hours and during an orthostatic challenge will be converted to text files for analysis with heart rate variability software (Hearts v7, Heart Signal Co., Oulu, Finland). Editing of the R-R interval time series is to be performed by a single investigator. All electrocardiogram signals will be manually scanned for ectopic or non-sinus beats, which will be deleted from the time series. Ninety percent of data are needed for inclusion of short-term heart rate variability recordings and 80% of data are needed for inclusion of long-term 24-hour recordings. Time domain heart rate variability analyses include heart rate, standard deviation of normal-to-normal intervals the root square mean of successive differences. The heart rate variability spectrum will be computed with the non-parametric fast Fourier transform method. Ultra low (0.0-0.003 Hz) and very low frequency powers (0.003-0.04) will be examined for 24-hour data and low (0.04-0.15 Hz) and high frequency powers (0.15-0.4 Hz) and the low-to-high frequency power ratio will be examined for both 24-hour and short-term data. A Poincaré plot will be formed by plotting each R-R interval against the following one to create a scatter plot. The standard deviation of the width and length will be calculated. The detrended fluctuation analysis method will be used to examine fractal characteristics of heart rate fluctuations. The root-mean square fluctuations of integrated and detrended data will be measured in observation windows and then plotted against the size of the window on a log-log scale. α_1_ will be calculated from the slope of the line (from 4-11 beats). Approximate entropy quantifies the regularity of time series data by calculating the likelihood that runs of patterns that are close will remain close on the next incremental comparison. A greater value represents greater unpredictability in a system. Approximate entropy will be calculated from 500 beats and will be computed with length, m = 2 and tolerance, r = 20%.

#### *Vascular data processing*

Systolic and diastolic radiuses along with pulse pressure will be entered into equations to calculate arterial compliance and distensibility [38]. Flow mediated dilation will be calculated as the maximal percentage increase in vessel diameter above baseline and percent change, absolute change and baseline diameter will be reported [39].

### Statistical analysis

#### *Outcome measures*

Clinic systolic blood pressure was chosen as the primary outcome measure.

Secondary outcomes included other metabolic syndrome, clinical and novel cardiometabolic risk factors. Scores from behavioural and health status questionnaires were also included in secondary outcomes.

Tertiary outcomes were mobile health variables including biometrics, activity and compliance to the monitoring protocol.

#### *Sample size calculation*

The sample size was based on differences in mean change between the intervention and active control groups in systolic blood pressure (primary outcome measure) at 12-weeks. The sample size calculation assumed 80% power and two-sided significance level of 0.05. We estimated that the smallest difference between groups in mean change that would be expected was 6 mmHg and we assumed a common standard deviation of 12 mmHg. Therefore, it was determined that 63 participants would be required per group. By assuming a 15% drop-out rate, this would increase our required sample size to 73 participants per group. Since we are using an Analysis of Covariance (ANCOVA) to perform data analysis (primary analysis), this sample size estimation may be considered conservative.

Data will be analysed following the Intent-to-Treat approach. Analyses will include all safety evaluable patients who have both baseline and valid post-baseline data. In instances where a participant may not have completed the final visit of the protocol but was subjected to the above procedures before study termination, a last observation carried forward approach will be used for analysis. Baseline data will not be carried forward.

For all continuous outcome variables, distributions will be examined to assess normality and presence of outliers; transformations will be undertaken as appropriate. For categorical outcome variables, frequency tables will be examined to ensure there are no out-of-range values.

Descriptive statistics will be computed for baseline demographic characteristics and for all outcome measures, at each time point. Specifically, means and standard deviations will be calculated for continuous variables and frequency counts and percentages will be calculated for categorical variables.

#### *12-week analyses*

For all continuous outcomes, ANCOVA will be used to examine differences in mean change between and within groups, while adjusting for baseline levels of the outcome of interest. For categorical outcomes, binary variables will be derived and we will use logistic regression to model the odds of improvement in either the intervention or active control group, while adjusting for baseline levels of the outcome of interest.

#### *52-week analyses*

The 12-week analyses will be extended to include data collected at all time-points. Linear mixed models will be used for continuous outcomes. Within these models, the group variable will indicate differences between groups at baseline, the time variable will indicate change over time in the reference (i.e., active control) group, and the group × time variable will indicate whether change over time differs between groups. Contrast statements will be used to determine change over time in the intervention group.

A similar approach will be followed for categorical variables; however, generalized linear (logistic) mixed models will be used instead. Interpretation of study results will be primarily based on estimation and associated 95% confidence intervals [40]. Analyses will be performed using R 3.0.1 [41].

## Discussion

This study investigated the effects of an exercise prescription supported by mobile health technology compared to an exercise prescription alone to improve metabolic syndrome and global cardiometabolic risk factors in rural community-dwelling adults. It was hypothesized that the primary outcome systolic blood pressure would be reduced to a greater extent in the mobile health-supported intervention group at 12-weeks and that this improvement would be better maintained in the intervention group for the remainder of the year-long trial. Similar improvements in secondary clinical outcomes as well as more novel cardiometabolic risk parameters including autonomic and vascular function were expected.

Interventions aimed at preventing chronic disease are important in rural communities with increased cardiometabolic risk and reduced access to healthcare. This study included adults from a rural community with greater prevalence of obesity, diabetes and hypertension than the provincial average [42] and reduced access to healthcare compared to urban communities [20]. Thus, it was considered an ideal setting for an intervention leveraging novel mobile health technologies to promote increased physical activity to reduce cardiovascular risk.

Exercise is known to have positive health benefits. The effects of exercise on individual metabolic syndrome risk factors have been reviewed extensively [4,43,44]. One meta-analysis examined the effects of exercise on metabolic syndrome risk factors in studies on populations with metabolic syndrome [6]. Of the studies included in the meta-analysis, training interventions were 8 to 52 weeks duration, frequency of two to five sessions per week for 40-60 minutes per session at moderate to high intensity, with the exception of one study which examined low intensity exercise. Endurance exercise training reduced waist circumference, systolic blood pressure and diastolic blood pressure and increased high density lipoprotein cholesterol, with no changes in fasting plasma glucose or triglcerides [6]. However, baseline fasting plasma glucose was within the normal range. Improvements would be expected if fasting plasma glucose was outside the normal range, as exercise improved HbA_1c_ in patients with type 2 diabetes mellitus [45]. Additionally, lifestyle interventions have been shown to improve autonomic [23,46-48] and vascular function [49,50]. Importantly, improvements in heart rate variability with lifestyle interventions were associated with lower risk of incident diabetes, independent of weight loss and physical activity [51].

Physician prescribed exercise has proved to effectively increase physical activity [13,14] and use of the STEP™ tool for fitness assessment and exercise prescription has been observed to improve the cardiometabolic risk profile of patients at risk [28]. A recent meta-analysis showed that STEP™ was the most effective prescription tool for increasing cardiorespiratory fitness, and it was suggested that this may be due to the inclusion of a training heart rate to ensure that the appropriate intensity of exercise was achieved [13]. Importantly, increased cardiorespiratory fitness reduced cardiovascular mortality in men [52]. However, with the current healthcare system, allied healthcare professionals may be more appropriate personnel to deliver STEP™. Hence, this trial will utilize a kinesiology-trained research assistant to assess fitness and prescribe exercise.

Intensive lifestyle intervention programs reduced the risk of developing type 2 diabetes mellitus in patients with prediabetes and improvements were maintained over a long-term follow-up; however, there was a strong relationship between reduced disease progression and continuance of lifestyle intervention behaviours [53]. This finding highlights the importance of post-program support for long-term maintenance. Implementation activities are important to translate research protocols into useable programs and a knowledge translation program including the construction of a national network of partners with interest in rural health and chronic disease management has been planned. A range of knowledge translation strategies will be utilized, including community consultations, physician and allied health provider presentations, scientific presentations and publications, town-hall meetings and gray literature publications among our end-users to ensure exposure and uptake among stakeholders.

This study was limited in that participants volunteered to enroll in the study. Thus, the study population consisted of highly motivated individuals who were interested in lifestyle improvements. Therefore, the findings may not be generalizable to a general clinic population, as many at-risk patients are not motivated to change their behaviours. Additionally, participants were limited to using study-provided smartphones to ensure data security. Whether differing devices and data providers will be similar is unknown.

In conclusion, this trial tested an intervention utilizing exercise prescription and mobile health monitoring technologies. Learnings from this study will be used to enhance the delivery of lifestyle interventions aimed at improving cardiometabolic risk, with the intention of reducing incident cardiovascular diseases and type 2 diabetes mellitus.

## Abbreviations

ANCOVA: Analysis of co-variance; STEP™: Step Test and Exercise Prescription.

## Competing interests

The authors have no competing interests to declare.

## Authors’ contributions

MS drafted the manuscript and participated in study design and data acquisition. SS was study coordinator, participated in study design and data acquisition and critically reviewed the manuscript. DG designed the plan for statistical analysis and critically reviewed the manuscript. RP was responsible for study conception and design. All authors read and approved the final manuscript.

## Pre-publication history

The pre-publication history for this paper can be accessed here:

http://www.biomedcentral.com/1471-2458/13/1051/prepub

## Supplementary Material

Additional file 1 **Online Supplement.** Target heart rate prescription according to fitness rating for men and women. Click here for file
