# Supplementary material for: A lifestyle intervention supported by mobile health technologies to improve the cardiometabolic risk profile of individuals at risk for cardiovascular disease and type 2 diabetes: study rationale and protocol
Source: BMC Public Health. 2013 Nov 7;13:1051. doi: 10.1186/1471-2458-13-1051 (PMC3922899; doi:10.1186/1471-2458-13-1051)
Supplement: Additional file 1 — Online Supplement. Target heart rate prescription according to fitness rating for men and women. [file 1471-2458-13-1051-S1.docx]

Online Supplement: Target heart rate prescription according to fitness rating for men and women.

**MEN:**

| **VO_2_max (ml/kg/min)** | **Rating** | **Heart Rate Prescription** |
| --- | --- | --- |
| **Aged 18-29 years** | | |
| ≤ 37 | Poor | 70% of max HR |
| 38-41 | Fair | 75% of max HR |
| 42-44 | Good | 70% of max HR |
| ≥ 45 | Excellent | 85% of max HR |
| **Aged 30-39 years** | | |
| ≤ 35 | Poor | 70% of max HR |
| 36-39 | Fair | 75% of max HR |
| 40-42 | Good | 80% of max HR |
| ≥ 43 | Excellent | 85% of max HR |
| **Aged 40-49 years** | | |
| ≤ 33 | Poor | 70% of max HR |
| 34-37 | Fair | 75% of max HR |
| 38-40 | Good | 80% of max HR |
| ≥ 41 | Excellent | 85% of max HR |
| **Aged 50-59 years** | | |
| ≤ 30 | Poor | 70% of max HR |
| 31-44 | Fair | 75% of max HR |
| 35-37 | Good | 80% of max HR |
| ≥ 38 | Excellent | 85% of max HR |
| **Aged 60 years +** | | |
| ≤ 26 | Poor | 70% of max HR |
| 27-30 | Fair | 75% of max HR |
| 31-34 | Good | 80% of max HR |
| ≥ 35 | Excellent | 85% of max HR |

**WOMEN:**

| **VO_2_max (ml/kg/min)** | **Rating** | **Heart Rate Prescription** |
| --- | --- | --- |
| **Aged 18-29 years** | | |
| ≤ 31 | Poor | 70% of max HR |
| 32-34 | Fair | 75% of max HR |
| 35-37 | Good | 80% of max HR |
| ≥ 38 | Excellent | 85% of max HR |
| **Aged 30-39 years** | | |
| ≤29 | Poor | 70% of max HR |
| 30-32 | Fair | 75% of max HR |
| 33-35 | Good | 80% of max HR |
| ≥ 36 | Excellent | 85% of max HR |
| **Aged 40-49 years** | | |
| ≤ 27 | Poor | 70% of max HR |
| 28-30 | Fair | 75% of max HR |
| 31-32 | Good | 80% of max HR |
| ≥ 33 | Excellent | 85% of max HR |
| **Aged 50-59 years** | | |
| ≤ 24 | Poor | 70% of max HR |
| 25-27 | Fair | 75% of max HR |
| 28-29 | Good | 80% of max HR |
| ≥ 30 | Excellent | 85% of max HR |
| **Aged 60 years+** |  |  |
| ≤ 23 | Poor | 70% of max HR |
| 24-25 | Fair | 75% of max HR |
| 26-27 | Good | 80% of max HR |
| ≥ 28 | Excellent | 85% of max HR |
